# Using a machine learning model to predict the development of acute kidney injury in patients with heart failure

**DOI:** 10.3389/fcvm.2022.911987

**Published:** 2022-09-07

**Authors:** Wen Tao Liu, Xiao Qi Liu, Ting Ting Jiang, Meng Ying Wang, Yang Huang, Yu Lin Huang, Feng Yong Jin, Qing Zhao, Qin Yi Wu, Bi Cheng Liu, Xiong Zhong Ruan, Kun Ling Ma

**Affiliations:** ^1^School of Medicine, Institute of Nephrology, Zhongda Hospital, Southeast University, Nanjing, China; ^2^John Moorhead Research Laboratory, Department of Renal Medicine, University College London (UCL) Medical School, London, United Kingdom; ^3^Department of Nephrology, The Second Affiliated Hospital, School of Medicine, Zhejiang University, Hangzhou, China

**Keywords:** heart failure, acute kidney injury, machine learning, prediction model, artificial intelligence

## Abstract

**Background:**

Heart failure (HF) is a life-threatening complication of cardiovascular disease. HF patients are more likely to progress to acute kidney injury (AKI) with a poor prognosis. However, it is difficult for doctors to distinguish which patients will develop AKI accurately. This study aimed to construct a machine learning (ML) model to predict AKI occurrence in HF patients.

**Materials and methods:**

The data of HF patients from the Medical Information Mart for Intensive Care-IV (MIMIC-IV) database was retrospectively analyzed. A ML model was established to predict AKI development using decision tree, random forest (RF), support vector machine (SVM), K-nearest neighbor (KNN), and logistic regression (LR) algorithms. Thirty-nine demographic, clinical, and treatment features were used for model establishment. Accuracy, sensitivity, specificity, and the area under the receiver operating characteristic curve (AUROC) were used to evaluate the performance of the ML algorithms.

**Results:**

A total of 2,678 HF patients were engaged in this study, of whom 919 developed AKI. Among 5 ML algorithms, the RF algorithm exhibited the highest performance with the AUROC of 0.96. In addition, the Gini index showed that the sequential organ function assessment (SOFA) score, partial pressure of oxygen (PaO_2_), and estimated glomerular filtration rate (eGFR) were highly relevant to AKI development. Finally, to facilitate clinical application, a simple model was constructed using the 10 features screened by the Gini index. The RF algorithm also exhibited the highest performance with the AUROC of 0.95.

**Conclusion:**

Using the ML model could accurately predict the development of AKI in HF patients.

## Introduction

Heart failure (HF) is the end stage of cardiovascular disease with a prevalence of around 1–2% in adults (1). HF patients are more likely to progress to acute kidney injury (AKI) with a poor prognosis (2). Studies have shown that more than 20% of HF inpatients would progress to AKI with a fatality rate of 4.1% (3, 4). Even mildly reversible AKI is associated with severe clinical outcomes, such as an increased risk of death (5, 6). Furthermore, HF has imposed a heavy financial burden on patients, with an annual cost ranging from $2,496 to $84,434 per patient (7).

Currently, it’s difficult for doctors to distinguish which patients will develop AKI. The diagnosis of AKI mainly depends on serum creatinine (Scr) and urine output. However, the elevation of Scr is usually delayed relative to the kidney injury, and Scr can be affected by muscle mass and metabolism (8). In addition, urine output is easily affected by drugs such as diuretics, and thus cannot reflect the kidney injury accurately. Therefore, some researchers have analyzed the risk factors of AKI, hoping to identify patients at high risk of AKI in advance. Fan et al. (9) employed a multivariate logistic regression method to reveal that age, diabetes, New York Heart Association (NYHA) classification, estimated glomerular filtration rate (eGFR), highly sensitive C-reactive protein (hs-CRP), and urinary angiotensinogen (uAGT) were independently associated with AKI development in HF patients. A meta-analysis also revealed that baseline chronic kidney disease (CKD), history of hypertension and diabetes, age, and diuretic use were significant predictors for AKI occurrence (3). In recent years, some researchers have adopted new biomarkers to predict the occurrence of AKI. Schanz et al. (10) examined urinary tissue inhibitors of metalloprotease-2 (TIMP-2) and insulin-like growth factor-binding protein 7 (IGFBP7) in 400 patients with HF. They found that urinary [TIMP-2] × [IGFBP7] was a promising marker for AKI risk assessment with high sensitivity and specificity. Although the above studies analyzed the risk factors of AKI, these studies adopted traditional strategies of developing prediction models and were not supported by big data. Therefore, more studies are needed to be carried out to verify the correctness of the above viewpoints.

Using machine learning (ML) algorithms is another strategy for establishing prediction models. ML is a subset of artificial intelligence (AI) in computer science. It is a discipline that focuses on how computers simulate human behaviors to acquire new knowledge (11). ML algorithms include decision tree, random forest (RF), support vector machine (SVM), logistic regression (LR), and K-nearest neighbor (KNN) (12). Compared with classical statistical methods, ML algorithms can explore the relationship between data and solve classification problems better (13, 14). Currently, the connection between ML and medicine is getting closer and ML has been adopted in the scope of diagnosis, risk stratification, and treatment (11, 15). Kimura et al. (16) utilized ML algorithms to analyze peripheral blood smears and developed an automated diagnostic model for myelodysplastic syndrome and aplastic anemia. In a multicenter study, Tomašev et al. (17) successfully developed a ML model to predict the occurrence of AKI, and stratified the risk of AKI to provide the possibility for the prevention of AKI. In addition, due to the support of ML algorithm in the treatment of anemia in hemodialysis patients, it not only reduced the use of erythropoietic-stimulating agent but also optimized anemia management (18). Overall, ML algorithms have made great contributions to improving the quality of healthcare.

Clinical studies often need the support of a large amount of data, and public databases can provide the required data. By analyzing the data in the database, researchers may draw valuable conclusions and help doctors make clinical decisions (19, 20). Medical Information Mart for Intensive Care-IV (MIMIC-IV) database contains clinical data on over 60,000 Intensive Care Unit (ICU) stays at the Beth Israel Deaconess Medical Center. Individuals who completed the test in PhysioNet have access to the database (certification number = 33449415) (21).

Machine learning algorithms have many advantages in the field of data processing. However, they are rarely used in AKI prediction in HF patients. Therefore, this study examined whether a ML-derived model for predicting AKI development would achieve high accuracy and guide AKI prevention.

## Materials and methods

### Study design and population

The data of HF patients hospitalized in the Cardiac Vascular Intensive Care Unit and Coronary Care Unit (CCU) in the MIMIC-IV database were retrospectively analyzed. With PostgreSQL 13, we installed the database on the computer. Next, demographics, clinical features, etc., of HF patients were extracted according to the corresponding codes. The inclusion criteria were as follows: 1) patients were older than 18 years old; 2) patients were diagnosed with HF according to the ICD code; 3) patients should have at least two Scr tests within the first 48 h of ICU admission; 4) For patients who were admitted to the hospital multiple times, the clinical data of the first hospitalization were selected. The exclusion criteria were as follows: 1) eGFR < 15 ml/min/1.73 m^2^ at the time of ICU admission; 2) patients received renal replacement therapy, including hemodialysis and peritoneal dialysis; 3) Patients whose Scr had risen ≥ 0.3 mg/dl before ICU admission during the hospitalization; 4) patients were diagnosed with heart transplantation, kidney transplantation, malignant tumor, and pregnancy; 5) patients who stayed in the ICU for less than 48 h. The primary endpoint was AKI, defined as the increase in Scr by ≥ 0.3 mg/dl within the first 48 h of ICU admittance (22). Because of inadequate data and probable changes in the urine output caused by medical therapy, urine output criterion was not employed to diagnose AKI.

After selecting patients, the demographic, clinical, and treatment data were extracted. A total of thirty-nine features were considered as AKI predictors. The eGFR was calculated by the CKD-EPI (Chronic Kidney Disease Epidemiology Collaboration) formula (23). In the analysis, missing data were replaced by mean or median according to data distribution. In addition, features with more than 30% missing data were not included.

### Establishment of the prediction model

**FIGURE 1:**
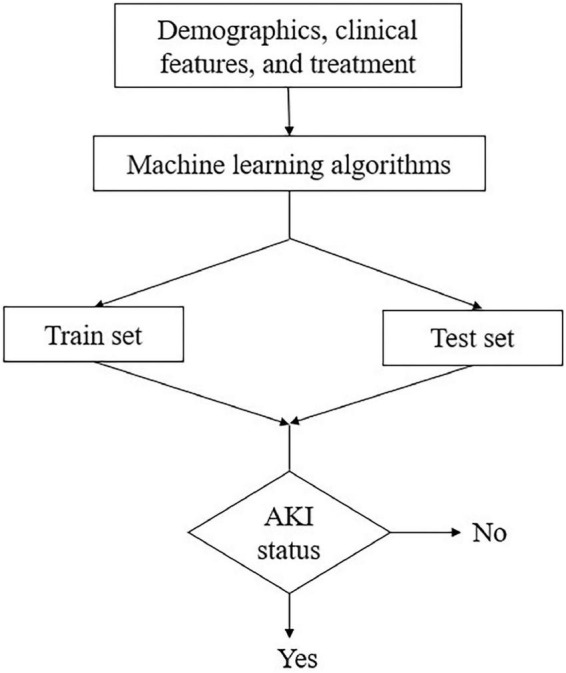
Process of establishing the prediction model. AKI, acute kidney injury.

Five ML algorithms: decision tree, RF, KNN, SVM, and LR were utilized to establish the model to predict the development of AKI. All the above features were incorporated into the ML model. A total of 70% of the dataset was randomly selected as the training set and the remaining 30% as the test set. The data in the training set was used to train the model, and the test set was used to examine the performance of the optimal model. The AKI status was classified as “Yes” or “No” (Figure 1). Accuracy, sensitivity, specificity, and the area under the receiver operating characteristic curve (AUROC) were used to evaluate the predictive performance of the model. The Gini index in the RF algorithm was calculated to rank the predictive value of features. To make the prediction model more concise and easier to use in clinical practice, a simple model with ten features selected by the Gini index was established. Python 3.7 was used to establish the model.

### Statistical analysis

For continuous variables, a *t*-test was used to compare the differences between two groups if they conformed to the normal distribution, otherwise rank-sum test was used. For categorical variables, the chi-square test was used for comparison. All tests of significance were 2-tailed, and the *P* < 0.05 was considered statistically significant. StataMP software (Version 14) was used for statistical analysis.

## Results

### Comparisons between acute kidney injury and non-acute kidney injury groups

**FIGURE 2:**
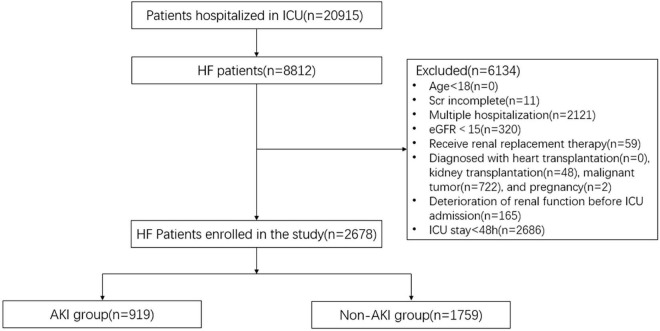
Consort flow chart. A total of 2,678 patients were selected from the database with 20,915 patients. ICU, intensive care unit; HF, heart failure; Scr, serum creatinine; eGFR, estimated glomerular filtration rate; AKI, acute kidney injury.

A total of 2,678 HF patients were engaged in the study (Figure 2). In our cohort, 919 HF patients progressed to AKI within the first 48 h of ICU admission. Males comprised 59.7 and 59.1% of AKI and non-AKI groups. Patients in the AKI group were significantly older than those in non-AKI group. Sequential organ function assessment (SOFA) score, Scr, and urea nitrogen were also higher in the AKI group. All comorbidities, including hypertension and diabetes, were highly related to AKI development. In addition, all treatments demonstrated significance between the two groups (Table 1).

**TABLE 1:** Clinical characteristics of HF patients.

Features	AKI (*n* = 919)	Non-AKI (*n* = 1759)	*P*-value
**Demographic/clinical characteristics**			
Age (year)	73 (63,81)	70 (59,80)	< 0.001
Male (%)	549 (59.7%)	1040 (59.1%)	0.759
Height (cm)	169 (163,175)	169 (165,178)	0.002
Weight (kg)	80 (67.8,95)	80.8 (67.9,96.3)	0.587
Respiratory rate (bpm)	16 (14,20)	18 (15,23)	< 0.001
Body temperature (°C)	36.6 (36.3,36.8)	36.6 (36.4,36.9)	< 0.001
Heart rate (bpm)	81 (74,91)	85 (74,97)	0.001
Systolic blood pressure (mmHg)	115 (99,132)	116 (102,131)	0.389
Diastolic blood pressure (mmHg)	59 (51,70)	63 (54,75)	< 0.001
SOFA score	8 (5,10)	5 (3,7)	< 0.001
Ventilation (%)	536 (58.3%)	678 (38.5%)	< 0.001
Diabetes (%)	435 (47.3%)	652 (37.1%)	< 0.001
CHD (%)	431 (46.9%)	748 (42.5%)	0.030
Hypertension (%)	246 (26.8%)	648 (36.8%)	< 0.001
Atrial flutter or atrial fibrillation (%)	535 (58.2%)	898 (51.1%)	< 0.001
COPD (%)	54 (5.9%)	162 (9.2%)	0.003
**Laboratory data**			
Scr (mg/dL)	1.2 (0.9,1.7)	1.1 (0.8,1.4)	< 0.001
eGFR (mL/min/1.73 m^2^)	54.9 (37.3,74.8)	65.3 (43.3,86.4)	< 0.001
Urea nitrogen (mg/dL)	25 (17,37)	22 (16,33)	< 0.001
WBC (K/μL)	12.3 (8.8,16.5)	11.2 (8.1,15)	< 0.001
Hemoglobin (g/dL)	9.4 (8,11.4)	10.7 (9,12.5)	< 0.001
Platelet (K/μL)	164 (120,227)	194 (145,250)	< 0.001
PT (s)	15.6 (13.9,18.5)	15 (13.1,17.5)	< 0.001
INR	1.4 (1.2,1.7)	1.4 (1.2,1.6)	< 0.001
PH	7.38 (7.33,7.44)	7.38 (7.36,7.43)	0.509
PaO_2_ (mmHg)	232 (116,347)	190 (86,272)	< 0.001
PaCO_2_ (mmHg)	41 (36,44)	42 (38,45)	< 0.001
Blood lactic acid (mmol/L)	1.9 (1.5,3)	1.9 (1.4,2.2)	< 0.001
Serum bicarbonate (mEq/L)	22 (20,24)	24 (22,27)	< 0.001
Serum potassium (mEq/L)	4.3 (3.9,4.8)	4.2 (3.8,4.6)	< 0.001
Serum sodium (mEq/L)	139 (136,141)	138 (136,141)	0.027
Serum calcium (mg/dL)	8.4 (8,8.8)	8.5 (8.1,8.9)	0.003
Serum magnesium (mg/dL)	2.2 (1.9,2.7)	2.1 (1.9,2.3)	< 0.001
Serum phosphate (mg/dL)	3.9 (3.3,4.8)	3.6 (3.1,4.2)	< 0.001
Blood glucose (mg/dL)	135 (108,178)	129 (108,168)	0.054
**Treatments**			
RAS inhibitor (%)	117 (12.7%)	442 (25.1%)	< 0.001
Diuretics (%)	806 (87.7%)	1329 (75.6%)	< 0.001
Digoxin (%)	30 (3.3%)	102 (5.8%)	0.004
β-receptor blocker (%)	459 (49.9%)	997 (56.7%)	0.001

Values are shown as median (interquartile range), absolute values, and percentages. SOFA, sequential organ function assessment; CHD, coronary heart disease; COPD, chronic obstructive pulmonary disease; Scr, serum creatinine; eGFR, estimated glomerular filtration rate; WBC, white blood cell; PT, prothrombin time; INR, international normalized ratio; PaO_2_, partial pressure of oxygen; PaCO_2_, partial pressure of carbon dioxide; RAS, renin-angiotensin system.

### Performance of the machine learning model

**FIGURE 3:**
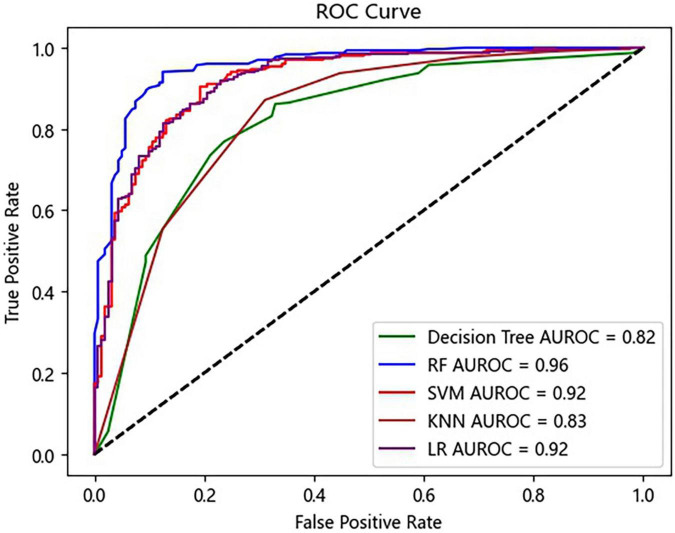
Receiver operating characteristic (ROC) curves of the prediction model. RF, random forest; SVM, support vector machine; KNN, K-nearest neighbor; LR, logistic regression.

Five ML algorithms were applied to predict the AKI status. Table 2 shows that the RF algorithm achieved the highest accuracy with 88.36%. In addition, the algorithms with the highest sensitivity and specificity were RF (96.04%) and LR (77.02%) (Table 2). The RF algorithm performed the best for the ML model with the AUROC of 0.96, which was better than 0.92 of SVM, 0.83 of KNN, 0.82 of the decision tree, and 0.92 of LR (Figure 3).

**TABLE 2:** Performance of the prediction model.

Algorithm	Accuracy (%)	Sensitivity (%)	Specificity (%)
RF	88.36	96.04	73.91
SVM	86.85	92.41	76.40
Decision tree	79.53	86.14	67.08
KNN	80.39	93.73	55.28
LR	86.42	91.42	77.02

RF, random forest; SVM, support vector machine; KNN, K-nearest neighbor; LR, logistic regression.

### Predictors of acute kidney injury status

**FIGURE 4:**
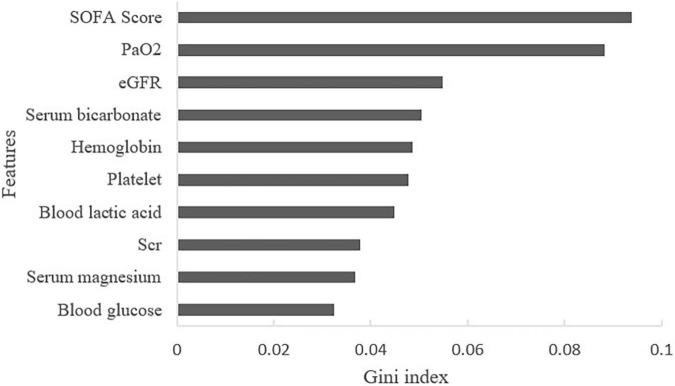
Contribution of features of AKI in HF patients (Top 10 displayed). SOFA, sequential organ function assessment score; PaO_2_, partial pressure of oxygen; eGFR, estimated glomerular filtration rate; Scr, serum creatinine.

By calculating the Gini index in the RF algorithm, the predictive value of features was ranked. The top ten predictors were: SOFA score, partial pressure of oxygen (PaO_2_), eGFR, serum bicarbonate, hemoglobin, platelet count, blood lactic acid, Scr, serum magnesium, and blood glucose (Figure 4).

### Establishment of a simple model

**FIGURE 5:**
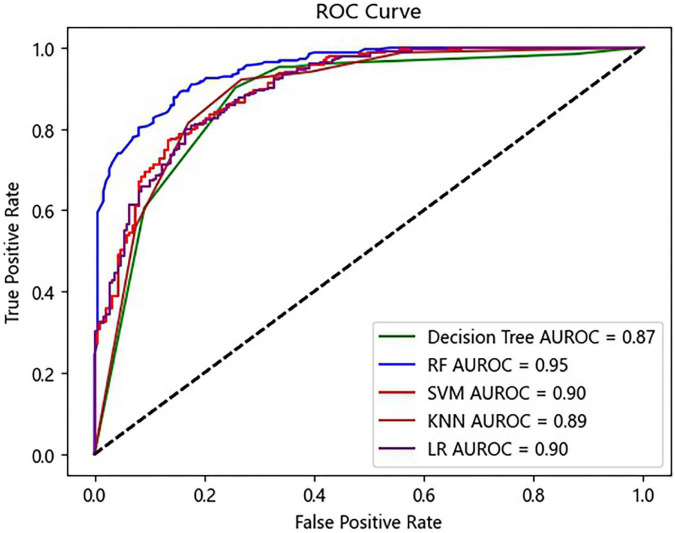
Receiver operating characteristic (ROC) curves of the prediction model using ten selected features. RF, random forest; SVM, support vector machine; KNN, K-nearest neighbor; LR, logistic regression.

According to the ten features selected by the Gini index, a simple model was established. Same as the prediction model using all 39 features, in the simple model, the RF algorithm achieved the highest accuracy with 87.07% (Table 3). In addition, the RF algorithm also achieved the highest sensitivity (92.52%), specificity (79.68%), and AUROC (0.95). Interestingly, the algorithms of KNN and decision tree outperformed the initial model with an improved AUROC (Figure 5).

**TABLE 3:** Performance of the simple model.

Algorithm	Accuracy (%)	Sensitivity (%)	Specificity (%)
RF	87.07	92.52	79.68
SVM	80.73	86.61	72.73
Decision tree	83.45	90.16	74.33
KNN	84.13	92.13	73.26
LR	81.63	88.19	72.73

RF, random forest; SVM, support vector machine; KNN, K-nearest neighbor; LR, logistic regression.

## Discussion

Different from previous studies, the strength of our study was the implementation of ML algorithms to predict AKI development (24, 25). Traditional approaches to constructing prediction models have made great contributions to assisting doctors in medical decision-making (26, 27). However, they have inherent drawbacks that may result in the omission of crucial predictors and correlations. Compared with traditional approaches, ML algorithms have great advantages in constructing prediction models, such as high accuracy in predicting heart disease (28, 29). According to our findings, the RF algorithm exhibited the highest performance among the five algorithms in predicting AKI. This is not surprising since the RF algorithm has advantages in processing high-dimensional data (30). In addition, the Gini index in the RF algorithm can reflect the predictive value of features, which facilitates the application of the prediction model in clinical practice. Therefore, from our point of view, RF algorithm should be preferentially adopted in clinical research, especially when analyzing high-dimensional data.

At present, HF patients are more likely to progress to AKI. Therefore, it is of great significance to analyze the risk factors of AKI and take corresponding treatment. The reported risk factors of AKI include age, baseline eGFR, NYHA classification, Kidney injury molecular-1, neutrophil gelatinase-associated lipocalin, urinary C-C motif chemokine ligand 14, etc. (9, 31, 32). However, some of these features are not routinely examined in clinical practice, which is not conducive to promotion. In this study, the features used in the prediction model are common and easy to obtain, which is also a major strength.

A total of 39 features were used to predict AKI in this study. Therefore, it is important to screen out the features related to the occurrence of AKI. Through the Gini index, we found that SOFA score, PaO_2_, and eGFR exhibited the highest predictive value. SOFA score can reflect the function of the nervous system, respiratory system, circulatory system, etc., and is used to monitor organ dysfunction. Currently, the SOFA score has been considered an excellent score to predict short-term mortality in life-threatening conditions (33). In critically ill patients, the SOFA score was thought to be an important predictor of AKI with the AUROC of 0.957 (34). In our study, patients with higher SOFA score were apt to progress to AKI, showing that the degree of organ dysfunction was associated with AKI. These findings suggest that SOFA scores should be routinely calculated in HF patients. Patients with high SOFA scores should be monitored and treated more aggressively.

In our study, patients who progressed to AKI had higher PaO_2_. This may be related to the high proportion of mechanical ventilation treatment in the AKI group. High PaO_2_ is related to oxidative stress, which is thought to be a pathogenesis of AKI (35, 36). Furthermore, Chen et al. (37) found that LPS-induced AKI in mice could be alleviated by inhibiting oxidative stress. Therefore, PaO_2_ may be a predictor of AKI. Currently, the relationship between hyperoxia and AKI is still inconclusive. Shen et al. (38) found that AKI was more common in patients with persistent hyperoxia than those with transient hyperoxia. In addition, according to an observational study by Bae, intraoperative hyperoxia was found to be strongly linked with the risk of AKI following cardiac surgery (39). Therefore, in clinical practice, we need to pay attention to the relationship between PaO_2_ and AKI, and further investigate the mechanism behind it.

Currently, eGFR is used to assess glomerular filtration function. In our study, we found that the eGFR of the AKI group was lower than that of the non-AKI group. It suggested that patients with kidney injury were more likely to progress to AKI. This finding was consistent with Tuukka’s study: lower baseline eGFR is an independent predictor of AKI (40). In a multicenter study, Patel et al. (41) performed a statistical analysis of more than 360,000 HF patients and found that 64% of them had eGFR < 60 mL/min/1.73 m^2^. In addition, they found that lower admission eGFR was associated with in-hospital mortality. Therefore, we recommend that eGFR should be calculated in each HF patient.

Finally, a simple model with ten selected features was established for the convenience of doctors. Compared with the prediction model using 39 features, the simple model was more usable and could also accurately predict AKI development. Interestingly, although there is less data in the simple model, the AUROC of KNN and decision tree algorithms were even higher. This phenomenon may be related to the removal of confounding factors. Therefore, screening predictors is also a key step when establishing the prediction model.

Our study had several limitations. First, in the MIMIC database, acute HF and chronic HF were not well distinguished. However, the severity of the two diseases was different, and the mechanisms that led to AKI were also different. We did not distinguish between the two diseases affected the correctness of the results to some extent. Second, this study was a single-center retrospective study without validation from other centers. Hence, high-quality randomized controlled trials are needed to confirm our findings.

## Conclusion

We successfully established a ML model to predict the development of AKI in HF patients. Among five ML algorithms, the RF algorithm exhibited the highest predictive performance. Our results provided the possibility for ML algorithms to guide AKI prevention in HF patients. Further studies are needed to verify whether our model can be applied to populations in other countries.

## Data availability statement

The original contributions presented in the study are included in the article/supplementary material, further inquiries can be directed to the corresponding author.

## Ethics statement

Ethical review and approval was not required for the study on human participants in accordance with the local legislation and institutional requirements. Written informed consent for participation was not required for this study in accordance with the national legislation and the institutional requirements.

## Author contributions

KM and WL: study design and writing – original draft. WL: data collection. WL, XL, TJ, and MW: statistical analysis. WL, YH, YLH, FJ, QZ, and QW: software. KM, XR, and BL: manuscript revision. All authors read and approved the final manuscript.

## Abbreviations

HF: heart failure
AKI: acute kidney injury
ML: machine learning
MIMIC-IV: Medical Information Mart for Intensive Care-IV
RF: random forest
SVM: support vector machine
KNN: K-nearest neighbor
LR: logistic regression
AUROC: area under the receiver operating characteristic curve
SOFA: sequential organ function assessment
PaO_2_: partial pressure of oxygen
eGFR: estimated glomerular filtration rate
Scr: serum creatinine
NYHA: New York Heart Association
hs-CRP: highly sensitive C-reactive protein
uAGT: urinary angiotensinogen
CKD: chronic kidney disease
TIMP-2: tissue inhibitor of metalloprotease-2
IGFBP7: insulin-like growth factor-binding protein 7
AI: artificial intelligence
ICU: intensive care unit
CKD-EPI: Chronic Kidney Disease Epidemiology Collaboration
CHD: coronary heart disease
COPD: chronic obstructive pulmonary disease
WBC: white blood cell
PT: prothrombin time
INR: international normalized ratio
PaCO_2_: partial pressure of carbon dioxide
RAS: renin-angiotensin system.
